# Supplementary material for: Time‑resolved multi-omic analysis of paclitaxel exposure in human iPSC‑derived sensory neurons unveils mechanisms of chemotherapy‑induced peripheral neuropathy
Source: Cell Death Dis. 2026 Feb 10;17(1):211. doi: 10.1038/s41419-026-08445-2 (PMC12921266; doi:10.1038/s41419-026-08445-2)
Supplement: Supplementary file 15 — Dataset 1. [file 41419_2026_8445_MOESM15_ESM.zip › CDD_Open_Data/05_Protein/02_Intersects_Disco_Plots/Andranik/72h.RNA_vs_protein.pdf]

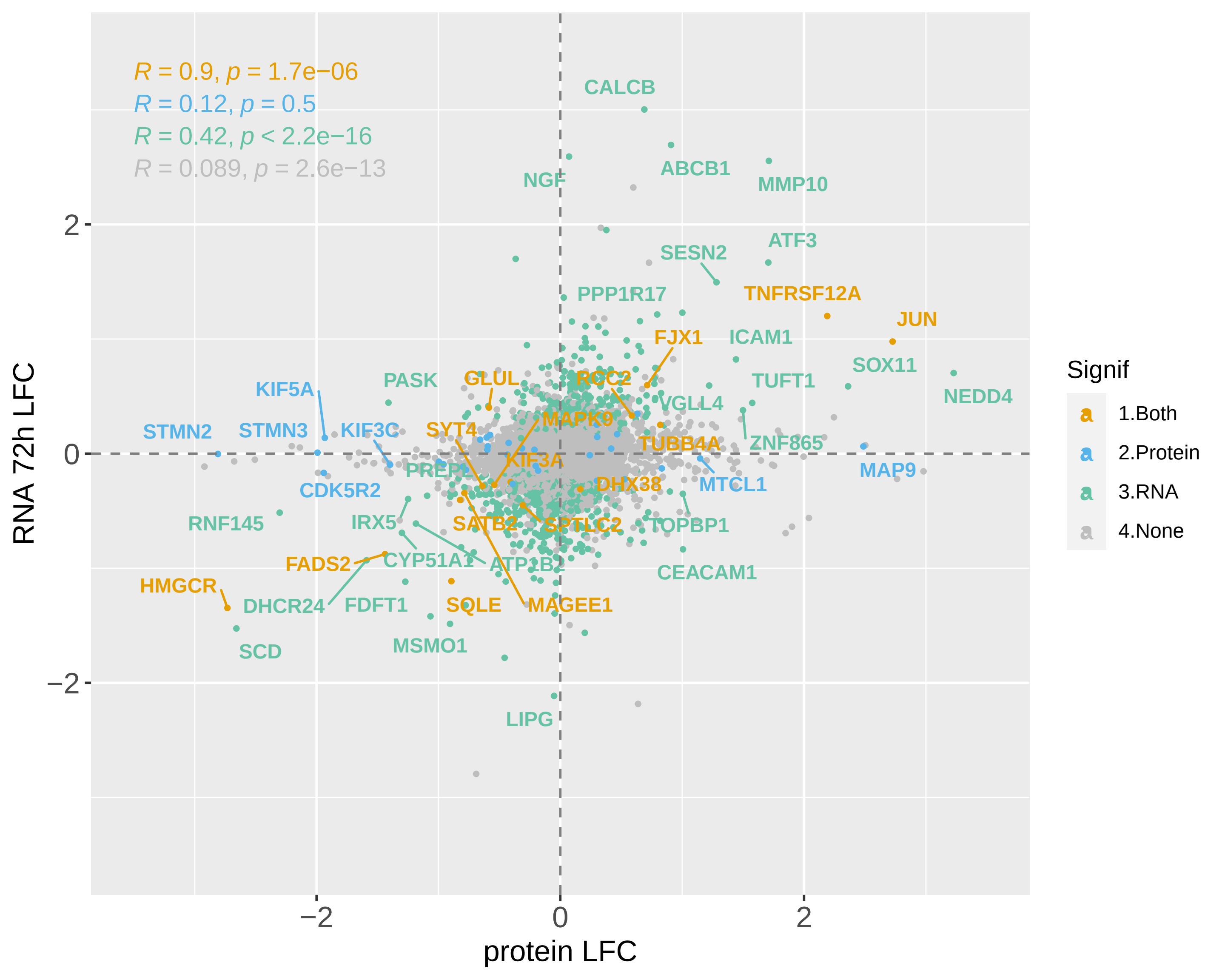

R = 0.9, p = 1.7e−06
R = 0.12, p = 0.5
R = 0.42, p < 2.2e−16
R = 0.089, p = 2.6e−13
CALCB
NGF
ABCB1
MMP10
ATF3
SESN2
PPP1R17
TNFRSF12A
JUN
FJX1
ICAM1
SOX11
NEDD4
TUFT1
GLUL
RCC2
PASK
KIF5A
VGLL4
MAPK9
STMN2
STMN3
KIF3C
SYT4
ZNF865
TUBB4A
MAP9
KIF3A
PREP
DHX38
MTCL1
CDK5R2
RNF145
IRX5
SATB2
SPTLC2
TOPBP1
FADS2
CYP51A1
ATP1B2
CEACAM1
HMGCR
DHCR24
FDFT1
SQLE
MAGEE1
SCD
MSMO1
LIPG
RNA 72h LFC
protein LFC
2
0
−2
Signif
1.Both
2.Protein
3.RNA
4.None
